# Case Report: A middle-aged woman with mediastinal follicular dendritic cell sarcoma complicated by paraneoplastic pemphigus

**DOI:** 10.3389/fonc.2025.1512156

**Published:** 2025-07-03

**Authors:** Yongchun Wang, Min Zhou, Feifei Wang, Zhiyan Hu, Yan Ruan

**Affiliations:** ^1^ Department of Otolaryngology, The Institute of The First Affiliated Hospital of Guangzhou University of Chinese Medicine, Guangzhou, China; ^2^ Guangzhou University of Chinese Medicine, The First Clinical Medical College, Guangzhou, China; ^3^ Department of Allergy, The Third Affiliated Hospital of Sun Yat-sen University, Guangzhou, China; ^4^ Department of Pathology, Nanfang Hospital, Southern Medical University, Guangzhou, Guangdong, China

**Keywords:** follicular dendritic cell sarcoma, paraneoplastic pemphigus, dental ulcer, differential diagnosis, multidisciplinary team consultation

## Abstract

As a relatively rare malignant entity, follicular dendritic cell sarcoma (FDCS) of the mediastinum currently suffers from insufficient clinical characterization, warranting more comprehensive studies to optimize therapeutic strategies. The initial symptoms of FDCS are often related to the respiratory system. It is particularly rare for the disease to present initially as recurrent ulcers in the oropharynx, which eventually led to a diagnosis of paraneoplastic pemphigus. Here, we report a case of a young female patient diagnosed with mediastinal FDCS associated with paraneoplastic pemphigus. The patient presented with recurrent pharyngeal ulcers and was diagnosed with mediastinal FDCS associated with paraneoplastic pemphigus 10 months later through imaging and pathological examinations.

## Introduction

Follicular dendritic cell sarcoma was first reported in 1986 by Monda in the form of a case report (1). It is a rare tumor associated with paraneoplastic pemphigus. Due to its distinctive growth pattern and cytological variability, it may be confused with a variety of tumors and even inflammatory processes (2–4). The initial symptoms of FDCS are diverse, and may manifest as myasthenia gravis (5), back pain, intestinal polyps (6, 7), recurrent oral ulcers (8–10), and other early symptoms (11–14). Paraneoplastic pemphigus is usually induced by lymphoproliferative diseases, and its classic clinical features include mucosal painful erosions appearing on the basis of latent or diagnosed tumors, as well as polymorphic skin lesions on the limbs and trunk. The diagnostic Criteria for Paraneoplastic Pemphigus (PNP) mainly present as: 1) Mucous membrane lesions with or without cutaneous involvement; 2) Concomitant internal neoplasm; 3) Evidence of anti-plakin autoantibodies, including various tests on transitional epithelium; 4) Acantholysis and/or lichenoid interface observed on histopathology, +/- necrotic keratinocytes; 5) direct immunofluorescence displaying intercellular and/or basement membrane staining. The diagnosis requires fulfillment of either all three primary criteria or any two of the first three criteria in combination with one criterion from items 4-5 (15). The combination of mediastinal dendritic cell sarcoma and paraneoplastic pemphigus is extremely rare, with only a limited number of studies reporting on this disease internationally (16–21). Our case report describes a patient with recurrent oral ulcers that were difficult to heal, who was ultimately diagnosed with mediastinal FDCS in association with paraneoplastic pemphigus.

## Case description

**Figure 1:**
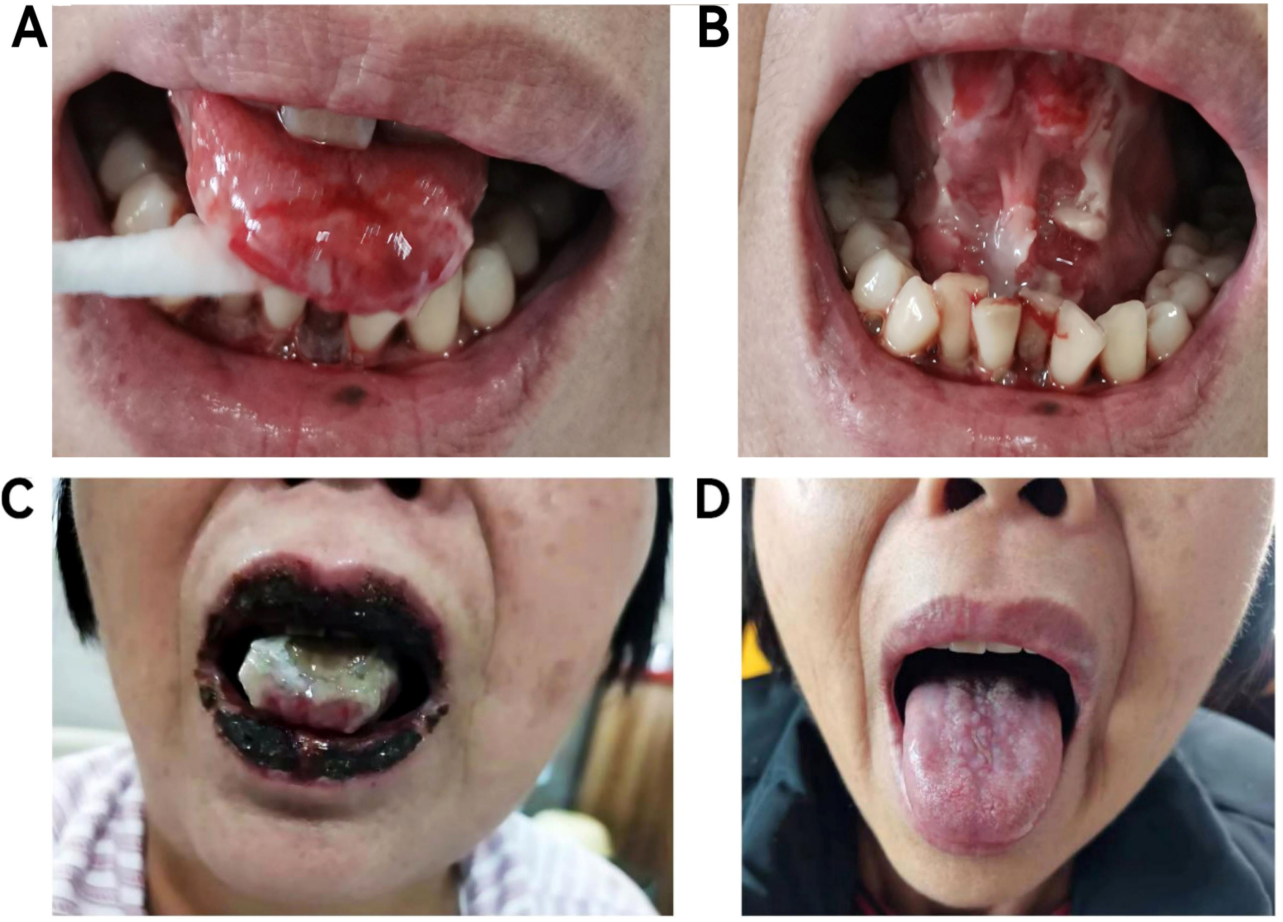
Changes in the patient’s tongue ulcers. **(A, B)** was taken 10 days earlier than **(C)**, showing that the ulceration of the tongue is evident and tends to bleed easily upon contact. **(C)** was taken in November 2022 before treatment, showing numerous brown crusts around the lips and multiple ulcers on the tongue, covered with yellowish-white secretions that were difficult to wipe off. **(D)** was taken in March 2024 after treatment, showing that the tongue had mostly returned to normal, with only minor granulation tissue proliferation.

**Figure 2:**
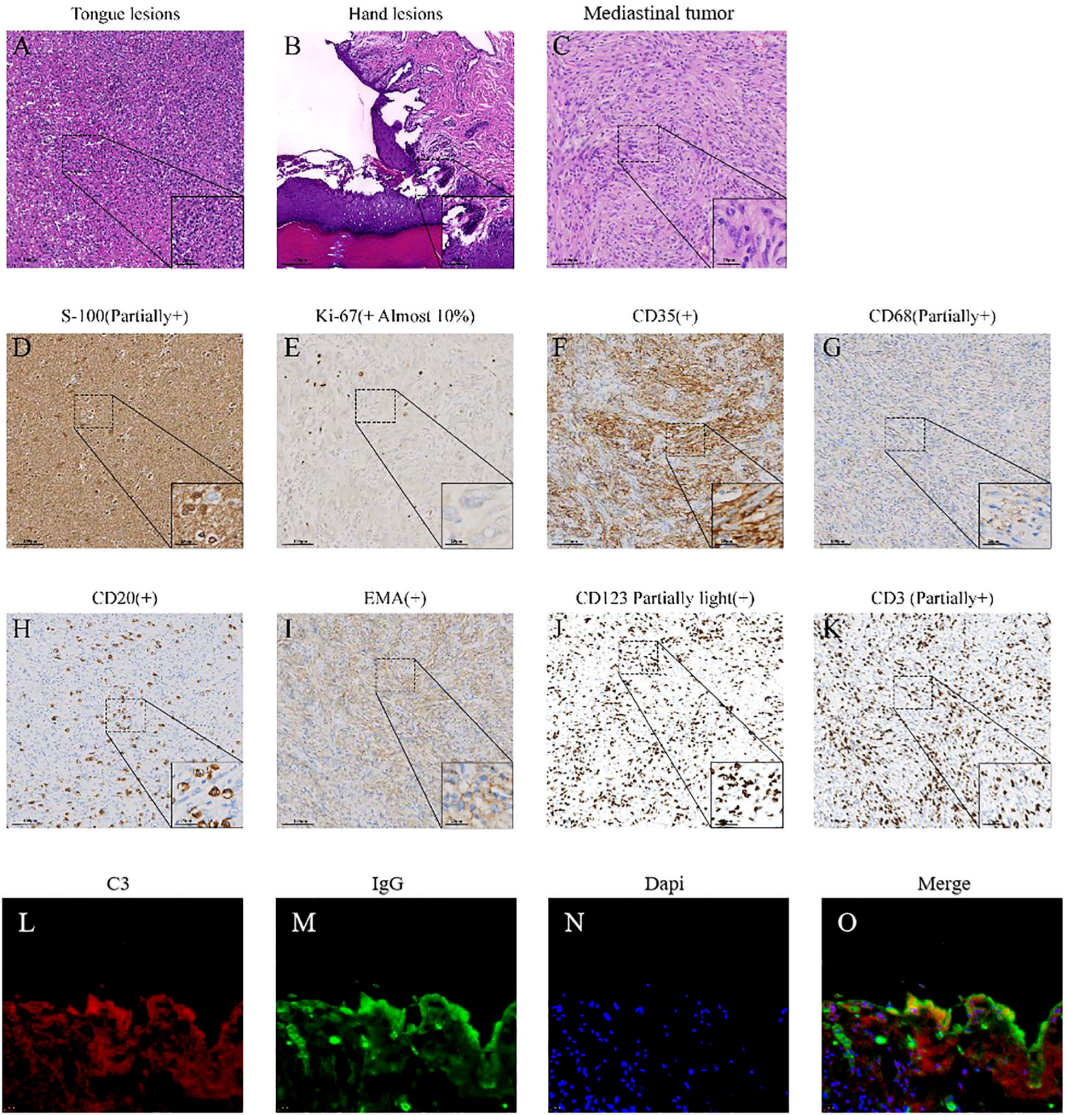
Pathological images of lesions. **(A)** The HE staining image of tongue lesions. Acute inflammatory exudate and multiple multinucleated giant cells can be seen in the figure. **(B)** The HE staining image of hand lesions. It shows mild hyperplasia of squamous epithelium with hyperkeratosis and focal hypokeratosis, local elongation of the skin foot, no liquid degeneration of the basal layer, no thickening of the basement membrane, local acantholysis and formation of epidermal bulls, the apex epidermis was generally normal, and local “gravestone” sign is observed, with hyperplasia of dermal papillary collagen fibers. Edema and infiltration of chronic inflammatory cells were observed around small vessels in the superficial dermis, and exudation of acute and chronic inflammatory cells was observed in the blister. **(C)** The HE staining of mediastinal mass. It can be seen that the tumor cells were arranged in spokes or swirls, and the tumor cells were fusiform and oval. **(D–K)** Immunohistochemical (IHC) staining of the mediastinal mass. **(L–O)** Immunofluorescence staining of the hand lesions showed IgG (green fluorescence) and C3 (red fluorescence) deposition predominantly within the epidermal compartment. The fluorescence intensity varied regionally, with focal areas of strong positivity alternating with zones of weaker signal, resulting in an overall irregular distribution pattern.

The patient (female, 58 years old) developed oral ulcers in January 2022, which did not improve significantly after self-treatment (oral cefaclor extended-release tablets 375 mg twice daily for one week). In March 2022, due to persistent erosions of the buccal mucosa, the patient sought care at several local hospitals and was initially diagnosed with lichen planus. In September 2022, the oral ulcers worsened, accompanied by hoarseness and tongue pain (**Figures 1A, B**). On September 6, 2022, the patient developed respiratory symptoms such as coughing, sputum production, and difficulty breathing, along with new-onset ulcers in the external genitalia, vesicles on the hands and feet, and crusted ulcers around the lips (**Figures 1C**, **2A**), as well as worsening conjunctival congestion, significant purulent discharge within the conjunctival sac, and cloudy corneas, indicating eye involvement. Histopathological examination of a blister from the dorsum of the hand demonstrated: suprabasal epidermal clefting (positive Nikolsky sign); mixed inflammatory cell infiltration around superficial dermal blood vessels (**Figure 2B**); DIF analysis of vesicular hand lesions (**Figure 2L**), which demonstrated: Immunofluorescence staining of the hand lesions showed IgG (green fluorescence) and C3 (red fluorescence) deposition predominantly within the epidermal compartment. The fluorescence intensity varied regionally, with focal areas of strong positivity alternating with zones of weaker signal, resulting in an overall irregular distribution pattern, confirming the tentative diagnosis of pemphigus vulgaris. The patient was treated for pemphigus vulgaris in the dermatology department from September 20, 2022, to October 17, 2022. Methotrexate combined with intravenous immunoglobulin (IVIG, 0.4 g/kg/day) was administered as treatment.

**Figure 3:**
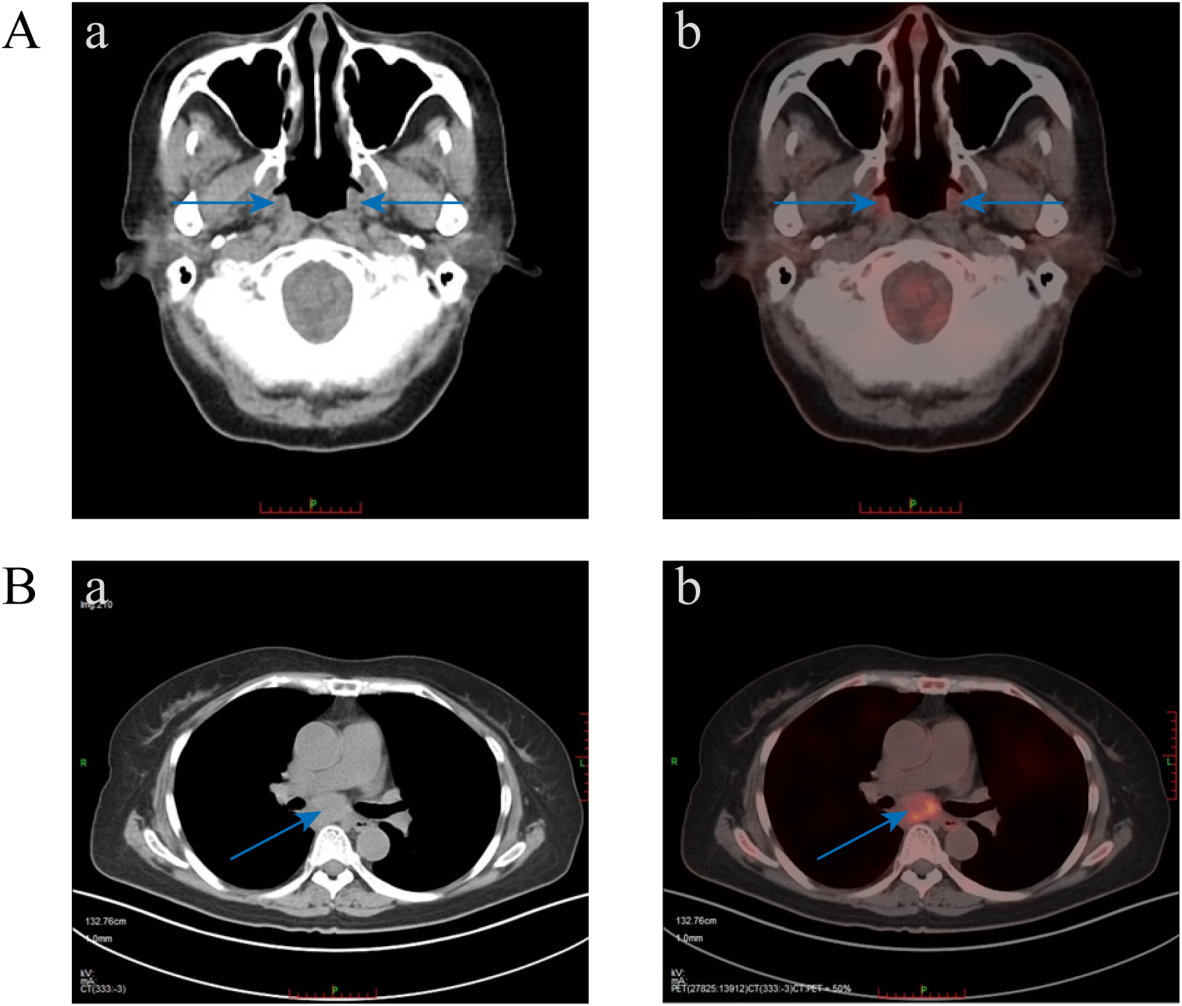
PET-CT images. **(A)** Diffuse mild increased metabolism of nasal alar, bilateral oral-buccal, bilateral periodontal, upper palate, oropharynx, laryngeal pharynx, and adjacent esophageal cervical mucosa was seen, which was considered to be caused by inflammation (blue arrow indicates increased metabolism area). **(B)** A mass of soft tissue in the mediastinum with increased metabolic heterogeneity.

On October 21, 2022, due to worsening cough, sputum production, and lack of improvement in the oral ulcers, the patient had a chest CT scan and the mediastinal mass was revealed. PET-CT revealed a mediastinal soft tissue mass measuring 4.7×2.8×5.9 cm with increased metabolic activity (SUVmax 7.3, SUVave 4.1) (**Figure 3**). The serological results demonstrated a weakly positive antinuclear antibody (ANA) with a titer of 1:80, exhibiting both nuclear speckled and cytoplasmic speckled patterns, while anti-double stranded DNA (anti-dsDNA) antibodies were negative. Complement 3 levels remained within normal limits without significant reduction. Notably, anti-desmoglein antibody testing revealed elevated anti-Dsg1 levels at 17.51 U/mL and anti-Dsg3 levels at 13.04 U/mL. Additionally, Epstein-Barr virus serology showed strongly positive EBV-VCA-IgG and EBV-NA-IgG titers (>50 AU/mL for both), indicative of past EBV infection.

Subsequently, on November 18, 2022, the patient underwent general anesthesia for thoracoscopic resection of the mediastinal mass. The final pathological diagnosis of the mediastinal mass was confirmed follicular dendritic cell sarcoma (**Figure 2C**), staged as pT2N0M0, with characteristic immunohistochemical profile demonstrating positivity for CD21 and CD35, while being negative for CD23, STAT6, and ALK, with the tumor exhibited a low proliferative index (Ki-67, 10%) (**Table 1**, **Figures 2D–K**). The mediastinal mass was completely excised with negative surgical margins, and showed no evidence of lymph node involvement in all examined nodal groups. Through multidisciplinary team consultation, the patient was ultimately diagnosed with: 1) follicular dendritic cell sarcoma (FDCS) and 2) paraneoplastic pemphigus (PNP), based on comprehensive evaluation of clinical manifestations (refractory oropharyngeal erosions + mediastinal tumor), laboratory findings (**Table 1**), histopathological examination of tongue, hand and mediastinal mass lesions, along with corresponding immunohistochemical profiles and immunofluorescence staining results (**Figure 2**).

**Table 1:** Laboratory findings of the patient.

Serological monitoring of pemphigus autoantibodies	Preoperative test	1-month postoperative test	2-month postoperative test	3-month postoperative test	6-month postoperative test	Reference range
Dsg1	17.15 U/ml	5.11 U/ml	5.04 U/ml	7.94 U/ml	13.04 U/ml	**Positive**≥20 U/ml; 14 U/ml≤**Weak Positive**<20 U/ml; **Negative**<14 U/ml
Dsg3	13.04 U/ml	2.38 U/ml	6.77 U/ml	3.53 U/ml	6.66 U/ml	**Positive**≥20 U/ml;7 U/ml<**Weak Positive**<20 U/ml; **Negative**<7 U/ml
**Peripheral Blood Immune Parameter Analysis**			**Index**	**Preoperative test**	**6-month postoperative test**	**Value**
			Ds-DNA	(-)	(-)	
			Jo-1	(+)1/80	(-)	Nuclear granular type: cytoplasmic granular type (1: 80)
			SS-B	(-)	(-)	
			AHA	(–)	(–)	
			Sm	(–)	(–)	
			UI-NRnp	(–)	(–)	
			ARPA/Rib-P	(–)	(–)	
			PCNA	(–)	(–)	
			AnuA	(–)	(–)	
			CENPB	(–)	(–)	
			PM-Scl	(–)	(–)	
			Sc1-70	(–)	(–)	
			SS-A	(–)	(–)	
			AMAM2	(–)	(–)	
			Ro52	(–)	(–)	
**Immunohistochemical results of mediastinal masses**			**Index**	**Positive**	**Negative**	**Value**
			CK		(–)	
			EMA	(+)		
			CK5/6		(–)	
			CD5		(–)	
			CD117		(–)	
			CD3	T cell(+)		
			CD20	B cell(+)		
			TdT		(–)	
			SOX-10		(–)	
			CD34		(–)	
			STAT-6		(–)	
			ALK		(–)	
			CD123	Partially light (+)		
			CD68	Partially (+)		
			CD163		(–)	
			CD21	(+)		
			CD35	(+)		
			Actin (SMA)		(–)	
			Desmin		(–)	
			S-100	Partially (+)		
			CD23		(–)	
			Ki-67	(+)		Almost 10%

The expression of pemphigus-related indicators Dsg1 and Dsg2, peripheral blood immune parameter analysis, and the immunohistochemical results of pathological sections.

**Figure 4:**
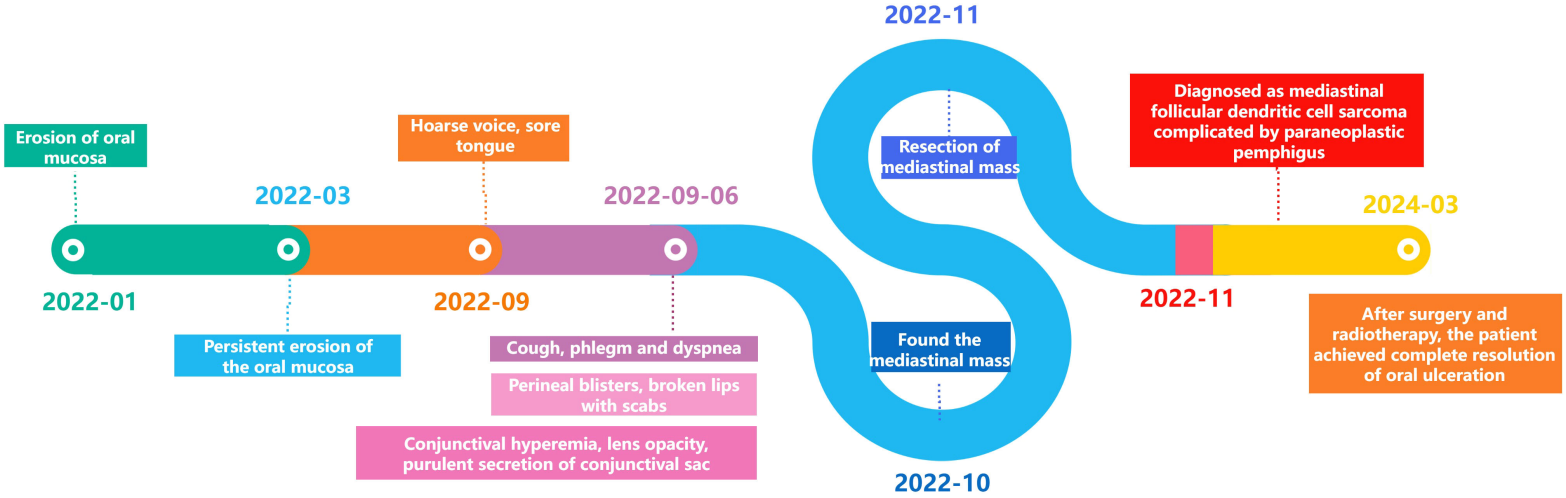
The entire disease progression of the patient.

Ten days post-surgery, the patient’s pemphigus antibodies (anti-Dsg1 13.04 U/mL, anti-Dsg3 6.66 U/mL) in the blood turned negative, but the oral ulcers worsened with a foul odor. Bacterial microscopy revealed Gram-positive cocci and Gram-negative bacilli, while fungal microscopy showed a small number of fungal spores. Bacterial culture indicated Klebsiella pneumoniae. After multidisciplinary consultation, the patient was treated with itraconazole, methylprednisolone, and symptomatic therapy, the oral ulcers significantly improved 15 days post-surgery. Subsequent postoperative radiotherapy and other related supportive treatments led to substantial healing of the oral ulcers and overall improvement in other symptoms. One year later (March 2024), the patient had no obvious ulceration in the oral cavity, only minimal scarring, and slight gingival swelling and tenderness (**Figure 1D**). The progression of the disease is shown in **Figure 4**.

## Discussion and conclusion

Diagnosing secondary tumor diseases is extremely complex, as they may present in various forms early on. Some may manifest as paraneoplastic syndromes of the endocrine system, leading to conditions such as inappropriate antidiuretic hormone secretion, hypercalcemia, Cushing’s syndrome, hypoglycemia, while others may present as paraneoplastic syndromes of the nervous system, including limbic encephalitis, paraneoplastic cerebellar degeneration, subacute peripheral sensory neuropathy (22–24). Additionally, they may manifest as paraneoplastic skin diseases and rheumatological syndrome symptoms such as acanthosis nigricans, pemphigus, polymyositis, Sweet’s syndrome, and other paraneoplastic skin conditions and rheumatic syndromes (25, 26).

The initial clinical manifestations of certain tumors can be remarkably insidious: bilateral periorbital cellulitis before lung cancer, dermatomyositis before breast cancer, reactive arthritis before thymoma, Kawasaki disease before early-stage stomach and esophageal cancer, etc. (27–29). This case of mediastinal FDCS complicated by PNP, initially manifesting solely as recurrent oropharyngeal mucosal lesions that presented clinically as nonspecific oral ulcers, underscores both the diagnostic elusiveness of early-stage paraneoplastic syndromes and the critical importance of maintaining high clinical vigilance for seemingly benign presentations.

This paradigmatic case also provides critical clinical insights that when faced with recurrent oral ulcers and mucosal erosion that are unresponsive to conventional treatment, we should initially rule out specific infectious diseases like diphtheria, streptococcal pharyngitis, scarlet fever, scarlet fever, and pharyngeal tuberculosis. Subsequently, non-infectious diseases, particularly immune system disorders, paraneoplastic disorders, and tumor diseases, such as lichen planus, Behçet’s disease, paraneoplastic pemphigus syndrome, systemic lupus erythematosus, should be considered. Given the difficulty of the exclusionary diagnosis in this stage, close monitoring and vigilance for any evolving symptoms are crucial.

The diagnosis of FDCS predominantly relies on pathological confirmation of tumor cells, complemented by the patient’s clinical symptoms and signs (30–32). For this patient, supplementary diagnostic workup is required to substantiate the FDCS diagnosis, with PDGFRB gene analysis being particularly indicative. Early detection and diagnosis of some FDCSs are challenging, often manifesting as paraneoplastic pemphigus syndrome with early symptoms. Biopsy of relevant crucial areas is crucial in early-stage inflammatory diseases. This case presented with dyspnea accompanied by pulmonary function tests demonstrating very severe mixed ventilatory dysfunction. Postoperative thoracic CT revealed pulmonary inflammatory changes with focal bronchial wall thickening, findings consistent with bronchiolitis obliterans. However, definitive diagnosis remains constrained by the absence of histopathological confirmation via bronchial tissue biopsy.

Additionally, FDCS and Castleman disease (CD) are distinct lymphatic disorders with divergent etiologies, pathophysiology, and clinical manifestations. FDCS, a rare malignancy of follicular dendritic cells, typically presents as a localized nodal or extranodal mass (e.g., liver, lung) with metastatic potential and expresses CD21/CD23/CD35 (31, 32). In contrast, CD is a benign lymphoproliferative disorder characterized by systemic symptoms (e.g., fever, weight loss) and IL-6-driven cytokine dysregulation. Accurate differentiation is essential for therapeutic decision-making.

For suspected paraneoplastic syndrome cases, a comprehensive diagnostic approach integrating laboratory tests (e.g., pemphigus antibodies), imaging (PET-CT), and pathological evidence (immunohistochemistry and immunofluorescence results) is essential (33). Future efforts should focus on optimizing early detection protocols for paraneoplastic syndromes through multidisciplinary collaboration and serial monitoring to reduce misdiagnosis of rare tumors and associated paraneoplastic syndromes. In summary, this case provides a diagnostic paradigm for FDCS-associated PNP with mucosal onset, highlighting the critical importance of multisystem evaluation and interdisciplinary cooperation in diagnosing rare paraneoplastic syndromes.

## Data Availability

The datasets presented in this study can be found in online repositories. The names of the repository/repositories and accession number(s) can be found in the article/supplementary material.
